# Supplementary material for: CNTNAP4 signaling regulates osteosarcoma disease progression
Source: NPJ Precis Oncol. 2023 Jan 4;7:2. doi: 10.1038/s41698-022-00344-x (PMC9813000; doi:10.1038/s41698-022-00344-x)
Supplement: Supplementary file 1 — Supplementary material [file 41698_2022_344_MOESM1_ESM.pdf]

Supplementary Figures

Supplementary Figure 1

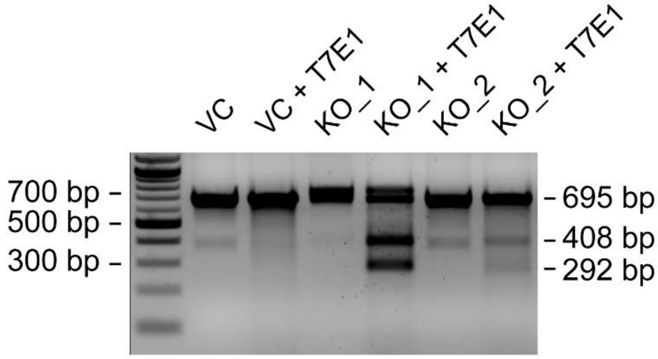

Supplementary Figure 1. T7 endonuclease I assay in two *CNTNAP4* knockout (KO) single cell clones. The cleaved PCR products correspond to 408 and 292 bp, respectively.

Supplementary Figure 2

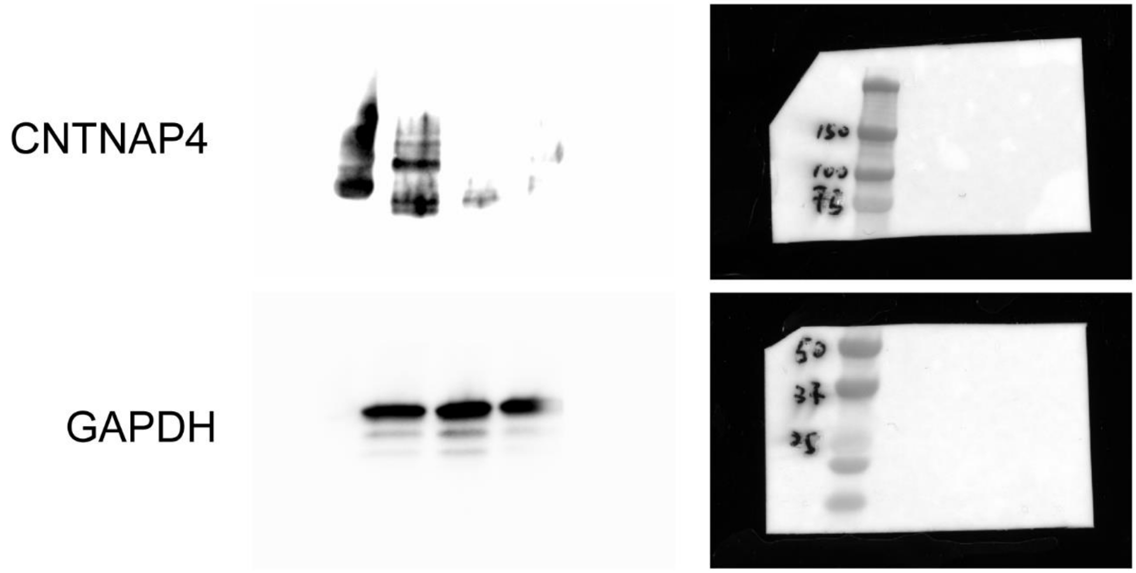

Supplementary Figure 2. Full-size blots of Figure 1c.

Supplementary Figure 3

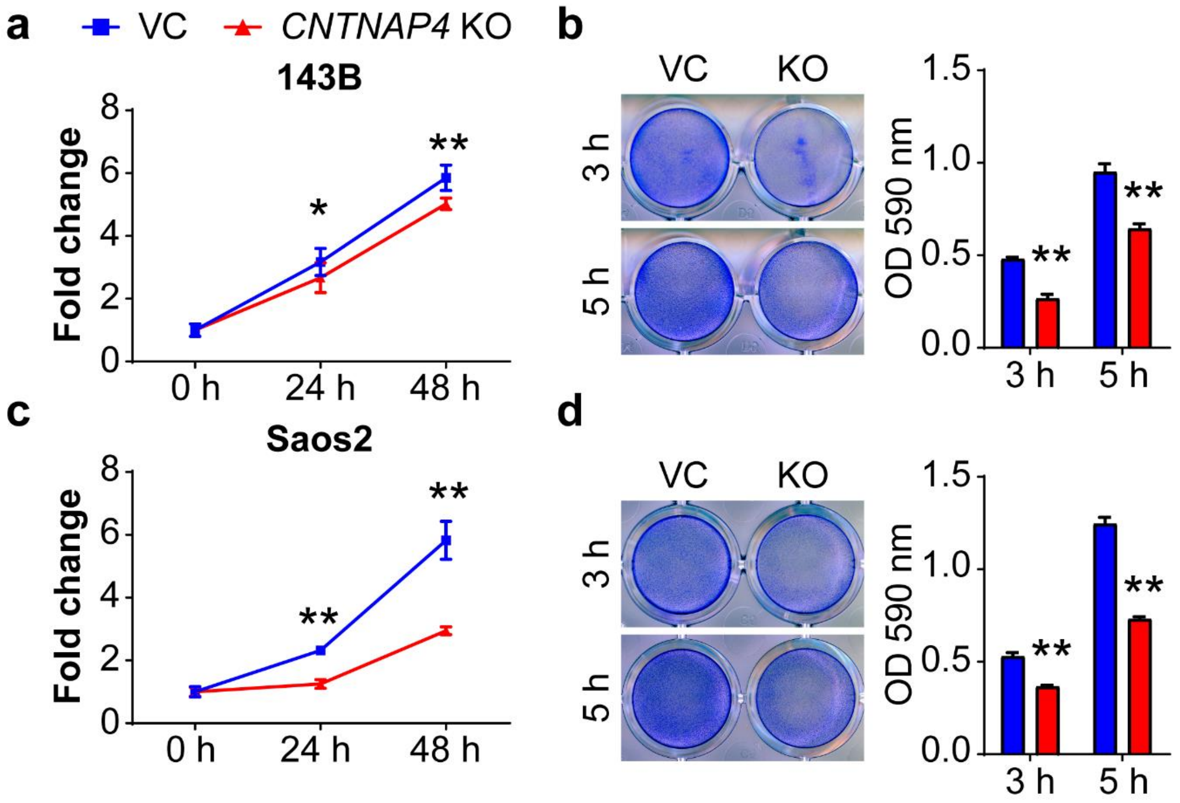

Supplementary Figure 3. CRISPR-Cas9 mediated *CNTNAP4* KO in polyclonal 143B and Saos2 cells. (a-b) Effects of CRISPR-Cas9 mediated *CNTNAP4* gene deletion in polyclonal 143B OS cells. (**a**) Proliferation (MTS) assay with or without *CNTNAP4* KO (0-48 h) (**b**) Attachment assay as assessed by crystal violet staining (left) and quantification (right) with or without *CNTNAP4* KO (3-5 h). (**c-d**) Effects of CRISPR-mediated *CNTNAP4* gene deletion in polyclonal Saos2 OS cells. (**c**) Proliferation assay as assessed by MTS with or without *CNTNAP4* KO (0-48 h). (**d**) Attachment assay as assessed by crystal violet staining (left) and quantification (right) with or without *CNTNAP4* KO (3-5 h). Data shown as mean ± 1 SD. All experiments performed in triplicates, with results from a single replicate shown. *P<0.05, **P<0.01.

Supplementary Figure 4

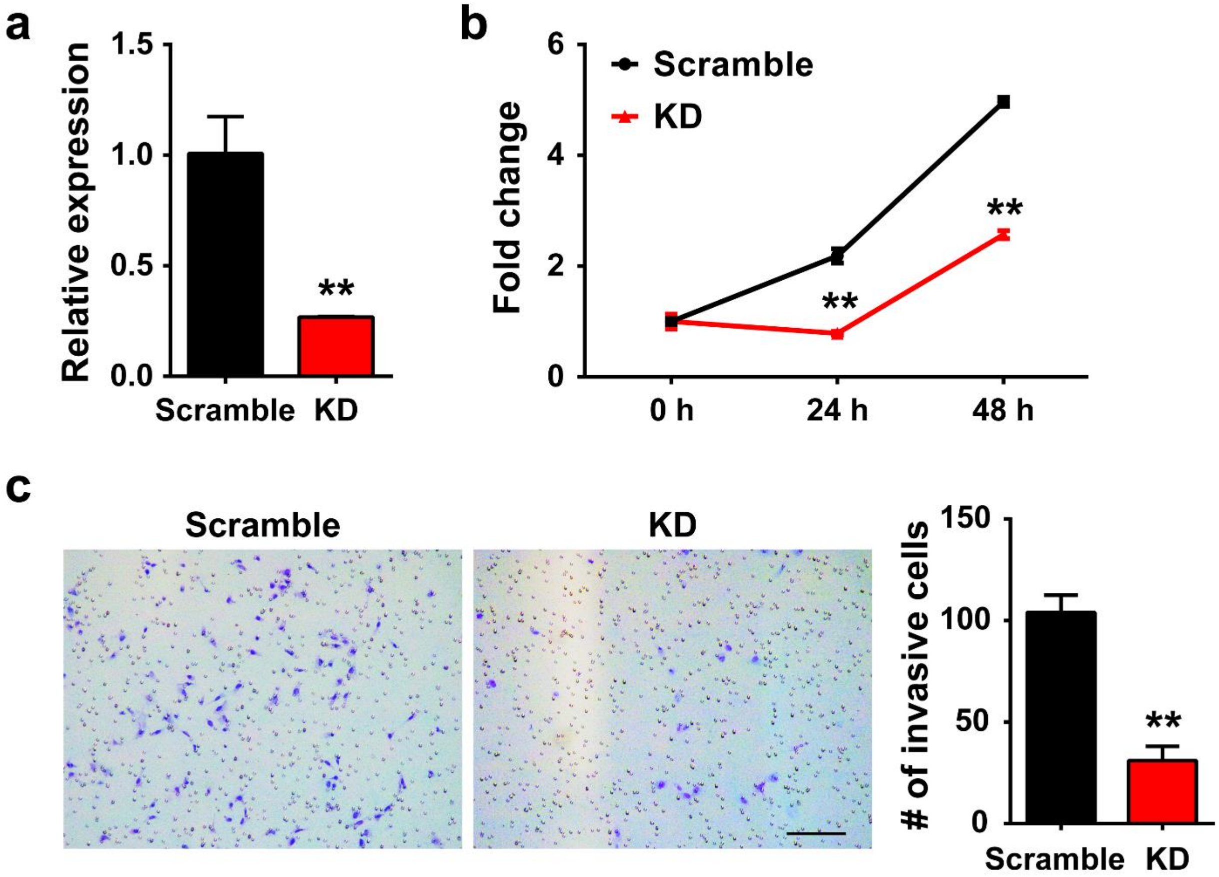

Supplementary Figure 4. SiRNA mediated *CNTNAP4* KD in 143B cells. Effects of SiRNA mediated *CNTNAP4* gene knockdown in 143B OS cells in comparison to scramble control. (**a**) Confirmation of *CNTNAP4* knockdown efficiency by qPCR, 48 h after siRNA treatment. (**b**) Proliferation (MTS) with or without *CNTNAP4* KD (0-48 h). (**c**) Transwell invasion assay with crystal violet staining, with or without *CNTNAP4* KD (22 h). Data shown as mean ± 1 SD. All experiments performed in triplicates, with results from a single replicate shown. $^{**}P<0.01$. Scale bar: 100 µm.

Supplementary Figure 5

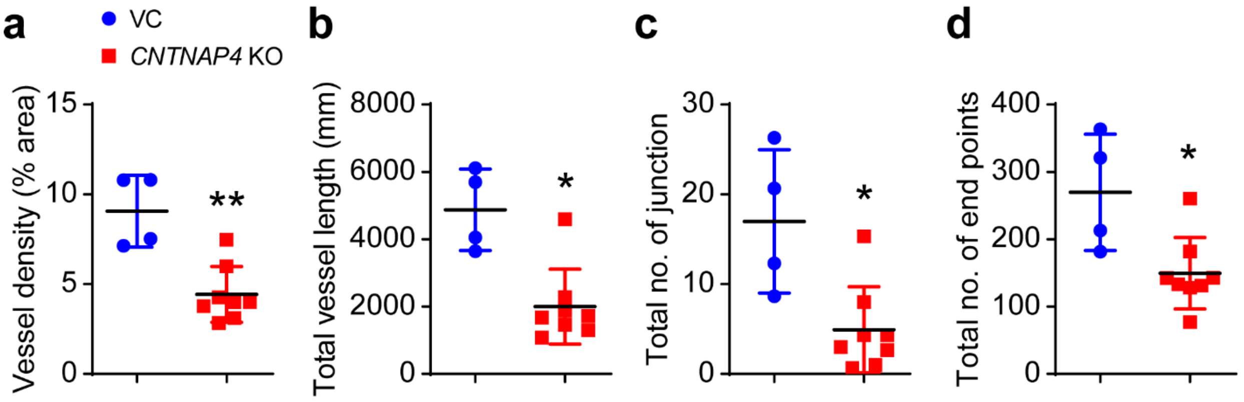

Supplementary Figure 5. Vascular histomorphometric analysis in *CNTNAP4* VC and KO xenograft explants. (**a**) Vessel density (**b**) total vessel length (**c**) total number of junctions, and (**d**) total number of endpoints. VC (n=4) and *CNTNAP4* KO (n=8) tumor explants analyzed. Data shown as mean ± 1 SD, ***P*<0.01, **P*<0.05.

Supplementary Figure 6

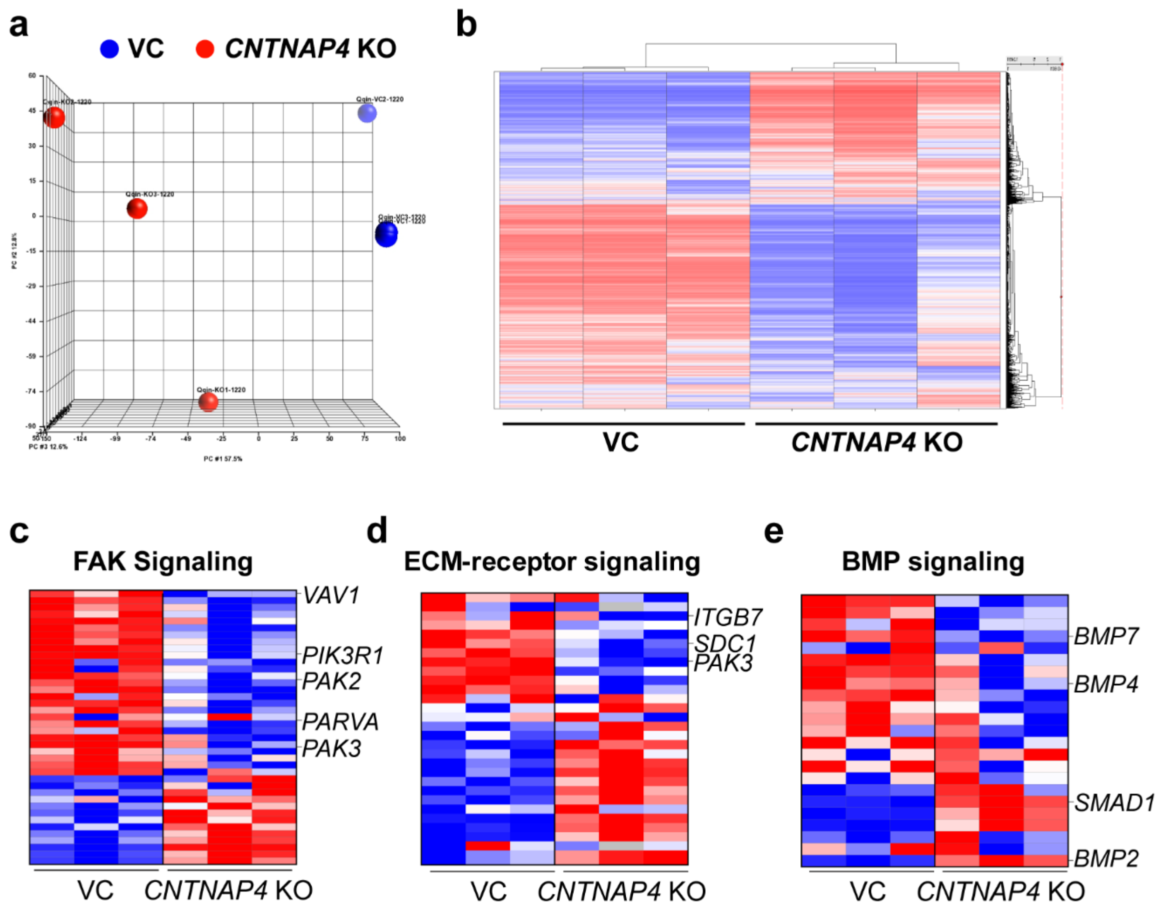

Supplementary Figure 6. Additional transcriptomic analysis of clonal 143B cells with or without *CNTNAP4* gene deletion. (**a**) Principal component analysis among VC and *CNTNAP4* KO 143B osteosarcoma cells. (**b**) Clustering heatmap of all 19,565 protein coding genes expressed among VC and *CNTNAP4* KO 143B cells. (**c**) Heatmap of representative FAK signaling pathway related genes. Note that *CNTNAP4* KO decreased expression level of certain key genes (*VAV1*, *PIK3R1*, *PAK2*, *PARVA*, *PAK3)* with implications in cancer progression. (**d**) Heatmap of representative ECM-receptor signaling. Note that *CNTNAP4* KO decreased expression level of certain key genes (*ITGB7*, *SDC1*, *PAK3*) with implications in cancer progression. (**e**) Heatmap of representative BMP signaling.

Supplementary Figure 7

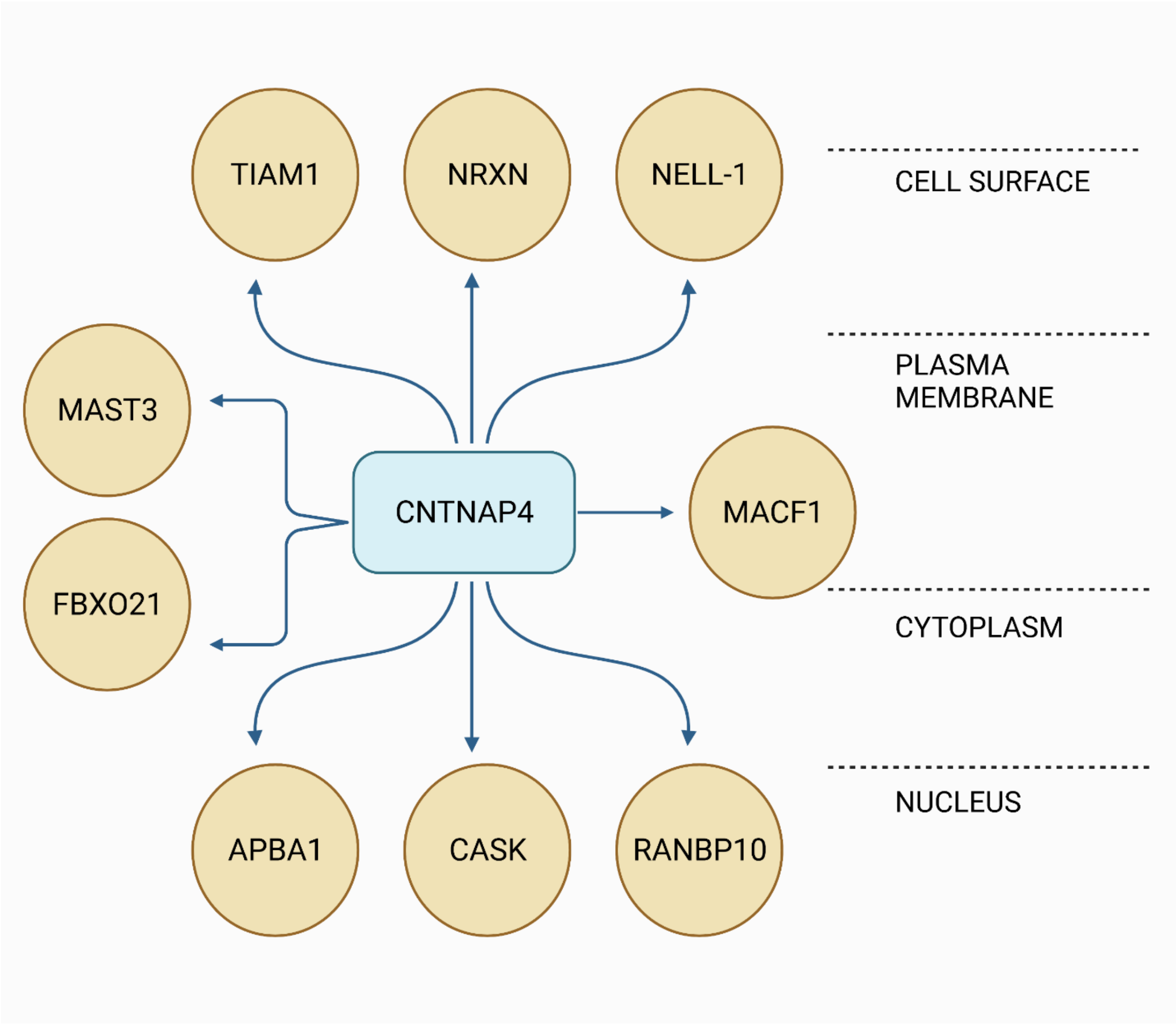

Supplementary Figure 7. Protein-protein interaction of CNTNAP4 network obtained using the STRING and Innate DB database. TIAM1, NELL-1, MACF1, MAST3, FBX021, NRXN, APBA1, CASK, and RANBP10 physically interact with CNTNAP4. Cartoon created with BioRender.com.

Supplementary Figure 8

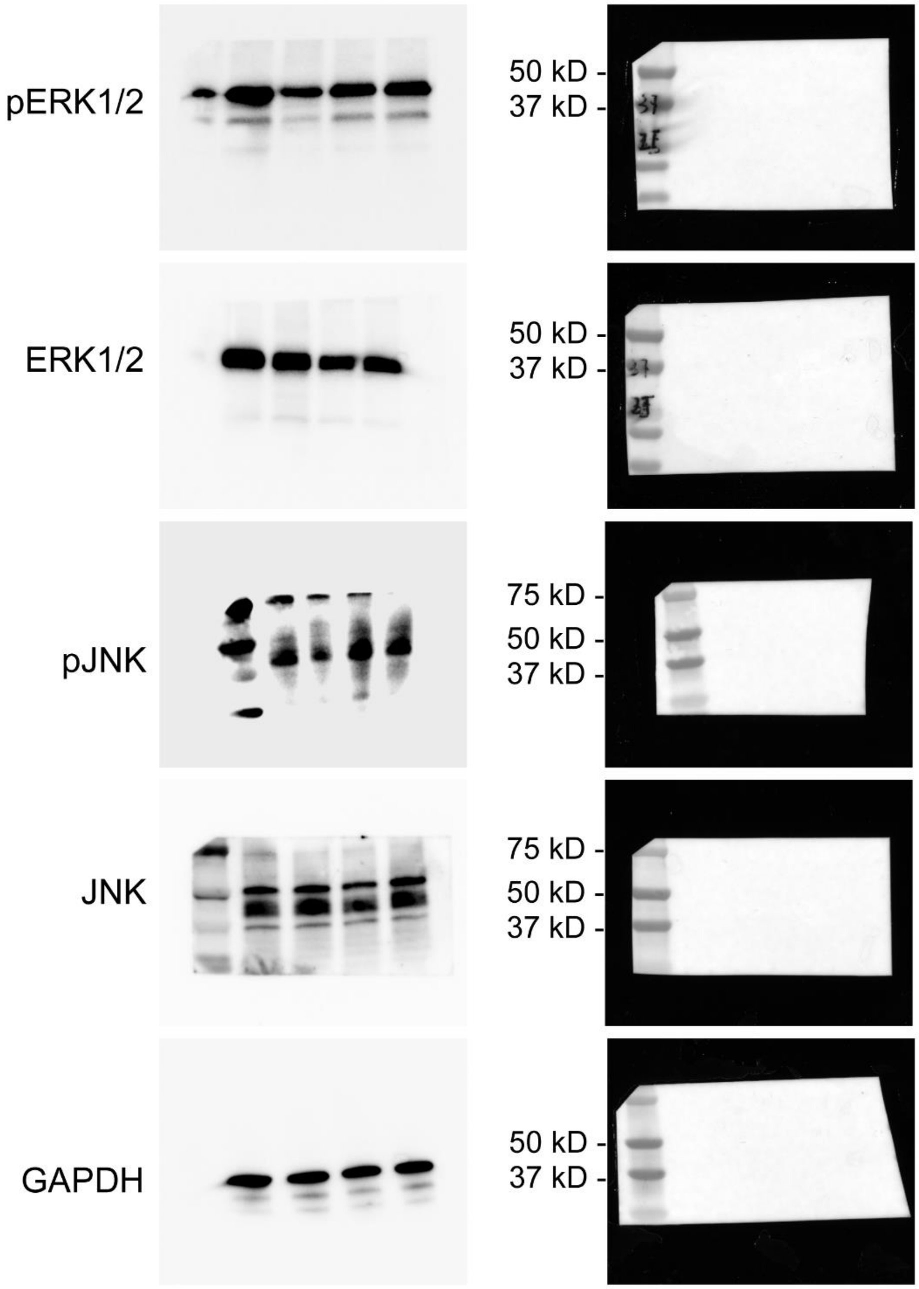

Supplementary Figure 8. Full-size blots of Figure 4g.

Supplementary Figure 9

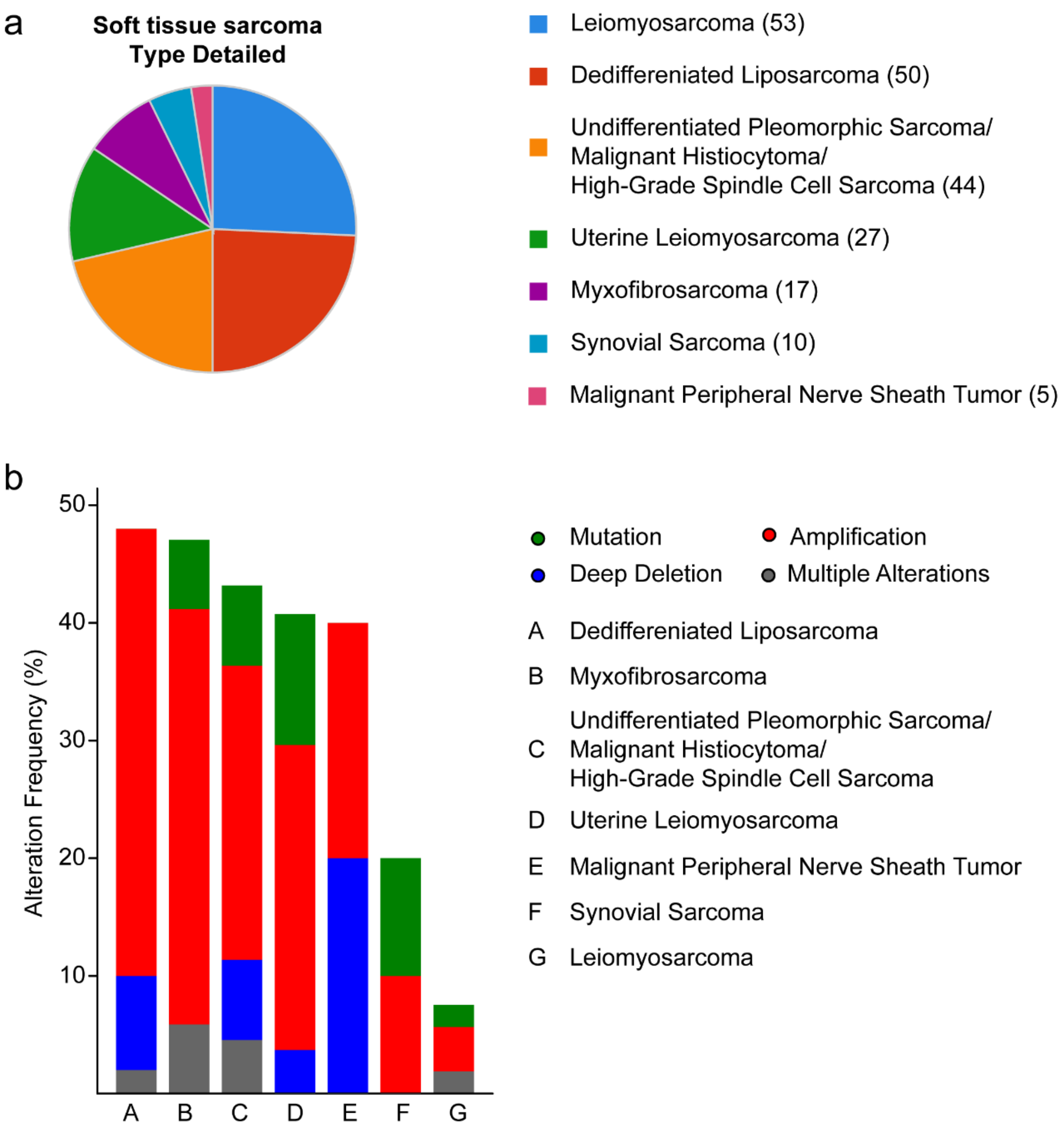

Supplementary Figure 9. Cancer subtypes and frequency of all mutations in *CNTNAP4* associated genes. (**a**) Pie chart shows percentage of patients in each soft tissue sarcoma subtype (n=206). (**b**) Bar graph represents the distribution of alteration types of *CNTNAP4* associated genes in each soft tissue sarcoma subtype, including amplification (red), deep deletion (blue), multiple alterations (grey), and missense mutation (green).

Supplementary Table 1. Enriched Gene Ontology (GO) terms related to cancer signaling.

**Downregulated GO terms_CNTNAP4 KO Vs VC**		
**GO term (Biological Processes)**	**Count**	***p*-value**
GO:0000165~MAPK cascade	90	0.004424
GO:1901796~regulation of signal transduction by p53 class mediator	65	1.73E-07
GO:0051056~regulation of small GTPase mediated signal transduction	61	1.30E-05
GO:0090263~positive regulation of canonical Wnt signaling pathway	53	0.001516
GO:0030036~actin cytoskeleton organization	53	0.031373
GO:0048013~ephrin receptor signaling pathway	36	1.86E-04
GO:0007409~axonogenesis	30	0.007502
GO:0008543~fibroblast growth factor receptor signaling pathway	27	0.04408
GO:0006977~DNA damage response, signal transduction by p53 class mediator resulting in cell cycle arrest	27	1.57E-04
GO:0048010~vascular endothelial growth factor receptor signaling pathway	23	0.031535
GO:0030514~negative regulation of BMP signaling pathway	23	0.037169
GO:0034446~substrate adhesion-dependent cell spreading	21	0.018607
GO:1904263~positive regulation of TORC1 signaling	10	0.031891
GO:0046328~regulation of JNK cascade	9	0.033764
GO:0035331~negative regulation of hippo signaling	9	0.002968
GO:0060338~regulation of type I interferon-mediated signaling pathway	7	0.006036
GO:0034333~adherens junction assembly	7	0.021732

Supplementary Table 2. Gene list used for heatmap.

MAPK signaling cascade	*FLT3, IRS1, YWHAB, NRK, RASGRF1, IRS2, PIK3CB, FGF1, FGF2, FGF5, FNTA, PSMD2, FNTB, PEA15, PBK, NEFL, PSMD1, NRTN, MAP3K9, MAP3K6, HRAS, MAP3K4, MAP2K4, KSR1, NRG1, FRS2, IL17RD, SPTB, DUSP7, PSMA6, PSMA3, PSMA4, PSMA2, RASA1, PSME3, MAPKAPK2, PSME2, RAPGEF2, PRKCQ, MAPKAPK5, RAF1, SOS1, CAMK2B, PSMD12, PSMD11, PPM1L, PSMD13, CUL3, TGFA, PIK3R1, RASAL2, ABHD17C, IL2RG, NLK, EGFR, PSMA7, RASGRP3, PSMB5, PABPN1, HSF1, MAP3K21, RIPK1, MAP4K5, PAK3, MARK3, MAP2K6, MAP3K2, ICMT, MAP3K1, DAB2IP, GRIN2B, FGF17, DLG1, PSMC6, ARTN, PSMC1, TAOK1, TAOK2, FGF18, NF1, LAMTOR2, GRB2, CALM1, FGF13, KBTBD7, MAP3K14, FGFR3, FGFR2, PTPN3, FGFR1*
Canonical Wnt signaling pathway	*OTUD5, PTPRU, GSK3B, GSK3A, CHD8, LIMD1, GLI3, PSMD2, PSMD1, LZTS2, ANKRD6, TLE4, TLE1, IGFBP4, IGFBP2, SOX13, SHISA3, TMEM170B, DKK1, LATS1, GREM1, PSMA6, SFRP1, PSMA3, PSMA4, PSMA2, PSME3, RAPGEF1, PSME2, ROR2, RBMS3, AMER1, PSMD12, PSMD11, PSMD13, CUL3, STK4, PSMA7, STK3, PSMB5, FRZB, G3BP1, APOE, FZD4, CAV1, DAB2IP, TMEM64, MAPK14, TPBGL, BICC1, PSMC6, PSMC1, CCDC88C, SNAI2, CTNNB1*
Fibroblast receptor signaling pathway	*TRIM71, FGF1, FGF2, FGF5, TIAL1, PTBP1, FLRT2, POLR2B, POLR2D, POLR2K, POLR2L, FGF21, GALNT3, NCBP2, FRS2, PTPN11, EXT1, FGF17, HNRNPM, HNRNPH1, FGF18, GRB2, FAT4, FGF12, FGFR3, FGFR2, FGFR1*
ECM-receptor interaction	*CD36 CD44, CD47, DAG1, GP1BA, GP1BB, GP5, GP6, GP9, HMMR, ITGA1, ITGA10, ITGA11, ITGA2, ITGA2B, ITGA3, ITGA4, ITGA5, ITGA6, ITGA7, ITGA8, ITGA9, ITGAV, ITGB1, ITGB3, ITGB4, ITGB5, ITGB6, ITGB7, ITGB8, SDC1, SV2A, SV2B, SV2C, VAV3, VCL*
FAK signaling pathway	*FAK, AKT1, AKT2, AKT3, BCAR1, CRK, CRKL, DOCK1, ILK, PAK1, PAK2, PAK3, PAK4, PAK5, PAK6, PARVA, PARVB, PARVG, PIK3CA, PIK3CB, PIK3CD, PIK3R1, PIK3R2, PIK3R3, PIP5K1A, PIP5K1B, PIP5K1C, PTK2, PXN, RAC1, RAC2, RAC3, RAP1A, RAP1B, RAPGEF1, RASGRF1, RHOA, ROCK1, ROCK2, VAV1, VAV2, TCF7L1, TCF7L2, WNT1, WNT10A, WNT10B, WNT11, WNT16, WNT2, WNT2B, WNT3, WNT3A, WNT4, WNT5A, WNT6, WNT7A, WNT7B, WNT8A, WNT8B, WNT9A, WNT9B, WNT5B, AXIN1, AXIN2*
CNTNAP4 interactors	*CNTNAP3B, APBA1, CNTNAP2, NELL1, CASK, TIAM1, AFDN, NRXN3, MACF1, NRXN2, CCDC184, FBXO2, MAST3, RANBP10*

Supplementary Table 3. CRISPR/Cas9 sgRNA and primer sequences.

**CRISPR/Cas9**	**Sequence**	
Knockout	5'- TTCCACGGAGAACTTAGCG-3'	
Negative control	5'-CGCGATAGCGCGAATATATT-3'	
**Primers**		
**Genes** (human)	**Forward** (5'-3')	**Reverse** (5'-3')
CNTNAP4 (T7EI)	GGCAGCATTTTTCCTTTCTTGC	CTCAGTGGCTAGACACCACC
CNTNAP4	TGCAAATGCAGACAGTGTTGT	CCCTTGGGGTTCCATTCCAAA
GAPDH	CTGGGCTACACTGAGCACC	AAGTGGTCGTTGAGGGCAATG

Supplementary Table 4. MAPK array protein targets layout.

<table>
<tr><th></th><th>A</th><th>B</th><th>C</th><th>D</th><th>E</th><th>F</th><th>G</th><th>H</th></tr>
<tr><td>1</td><td rowspan="2">POS</td><td rowspan="2">POS</td><td rowspan="2">NEG</td><td rowspan="2">NEG</td><td rowspan="2">Akt
(pS473)</td><td rowspan="2">CREB
(pS133)</td><td rowspan="2">ERK1
(pT202/Y204)
/ERK2
(Pt185/y187)</td><td rowspan="2">GSK3a
(pS21)</td></tr>
<tr><td>2</td></tr>
<tr><td>3</td><td rowspan="2">GSK3b
(pS9)</td><td rowspan="2">HSP27
(pS82)</td><td rowspan="2">JNK
(pT183)</td><td rowspan="2">MEK
(pS217/T221)</td><td rowspan="2">MKK3
(pS189)</td><td rowspan="2">MKK6
(pS207)</td><td rowspan="2">MSK2
(pS360)</td><td rowspan="2">mTOR
(pS2448)</td></tr>
<tr><td>4</td></tr>
<tr><td>5</td><td rowspan="2">p38
(pT180/Y182)</td><td rowspan="2">p53
(pS15)</td><td rowspan="2">P70S6K
(pT421/S424)</td><td rowspan="2">RSK1
(pS380)</td><td rowspan="2">RSK2
(pS386)</td><td rowspan="2">NEG</td><td rowspan="2">NEG</td><td rowspan="2">POS</td></tr>
<tr><td>6</td></tr>
</table>

Supplementary Table 5. List of antibodies used.

Antibody	Company	Catalog #	Use	Dilution
Rabbit anti-CNTNAP4	Biorbyt	orb544737	IHC, WB	1:100
Mouse anti-Human Nuclei	Millipore-Sigma	MAB1281	IF	1:200
Rabbit anti-CD31	Abcam	ab28364	IF	1:200
Rabbit anti-Ki67	Abcam	ab15580	IF	1:200
Goat anti-Mouse AF488	Abcam	ab150117	IF	1:1000
Goat anti-Rabbit AF488	Abcam	ab150077	IF	1:1000
Goat anti-Rabbit DyLight 594	Vector Laboratories	DI-1594	IF	1:1000
Goat anti-Rabbit, HRP	Invitrogen	32460	IHC	1:1000
Rabbit anti-p44/42 MAPK (Erk1/2)	Cell Signaling Technology	9102	WB, IF	1:100
Rabbit anti-Phospho-p44/42 MAPK (Erk1/2)	Cell Signaling Technology	9101	WB, IF	1:100
Rabbit anti-FGF	Cell Signaling Technology	61997	WB	1:100
Rabbit anti-JNK	Cell Signaling Technology	9252	WB	1:100
Mouse anti- Phospho-JNK	Cell Signaling Technology	9255	WB	1:100
Rabbit anti-GAPDH	Cell Signaling Technology	5174	WB	1:100
Anti-mouse IgG, HRP-linked Antibody	Cell Signaling Technology	7076	WB	1:2500
Anti-biotin, HRP-linked Antibody	Cell Signaling Technology	7075	WB	1:2500
Anti-rabbit IgG, HRP-linked Antibody	Cell Signaling Technology	7074	WB	1:2500
IF: Immunofluorescent staining; IHC: Immunohistochemistry; WB: Western Blot				
