# Supplementary material for: CNTNAP4 signaling regulates osteosarcoma disease progression
Source: NPJ Precis Oncol. 2023 Jan 4;7:2. doi: 10.1038/s41698-022-00344-x (PMC9813000; doi:10.1038/s41698-022-00344-x)
Supplement: Supplementary file 3 — REPORTING SUMMARY [file 41698_2022_344_MOESM3_ESM.pdf]

nature portfolio

Corresponding author(s): Aaron James
Last updated by author(s): Nov 7, 2022

Reporting Summary

Nature Portfolio wishes to improve the reproducibility of the work that we publish. This form provides structure for consistency and transparency in reporting. For further information on Nature Portfolio policies, see our Editorial Policies and the Editorial Policy Checklist.

Statistics

For all statistical analyses, confirm that the following items are present in the figure legend, table legend, main text, or Methods section.

n/a	Confirmed	
☐	☒	The exact sample size (n) for each experimental group/condition, given as a discrete number and unit of measurement
☐	☒	A statement on whether measurements were taken from distinct samples or whether the same sample was measured repeatedly
☐	☒	The statistical test(s) used AND whether they are one- or two-sided<br>*Only common tests should be described solely by name; describe more complex techniques in the Methods section.*
☐	☒	A description of all covariates tested
☐	☒	A description of any assumptions or corrections, such as tests of normality and adjustment for multiple comparisons
☐	☒	A full description of the statistical parameters including central tendency (e.g. means) or other basic estimates (e.g. regression coefficient) AND variation (e.g. standard deviation) or associated estimates of uncertainty (e.g. confidence intervals)
☐	☒	For null hypothesis testing, the test statistic (e.g. F, t, r) with confidence intervals, effect sizes, degrees of freedom and P value noted<br>*Give P values as exact values whenever suitable.*
☒	☐	For Bayesian analysis, information on the choice of priors and Markov chain Monte Carlo settings
☒	☐	For hierarchical and complex designs, identification of the appropriate level for tests and full reporting of outcomes
☐	☒	Estimates of effect sizes (e.g. Cohen's d, Pearson's r), indicating how they were calculated

Our web collection on statistics for biologists contains articles on many of the points above.

Software and code

Policy information about availability of computer code

Data collection	Digital images of these sections were captured using upright fluorescent microscope Leica DM6, Leica Microsystems Inc., Buffalo Grove, IL. Quantitative vascular morphometric analysis, a semi-automated, validated, open-source software Angiotool 0.6a, http://angiotool.nci.nih.gov.
Data analysis	cBioPortal for Cancer Genomics repository was utilized to reanalyze multi-omics data from the Cancer Genome Atlas [Adult Soft Tissue Sarcomas (TCGA, Cell 2017)]. Real Time PCR was performed using Quant Studio, Applied Biosystems v.1.4.3. Immunoblot was imaged using ChemiDoc Touch Imaging System (Bio-Rad, CA). For RNAseq, data analysis was performed using software packages including CLC Genomics Server and Workbench (RRID: SCR_017396 and RRID: SCR_011853), Partek Genomics Suite (RRID: SCR_011860), Spotfire DecisionSite with Functional Genomics (RRID: SCR_008858), and QIAGEN Ingenuity Pathway Analysis (IPA, RRID: SCR_008653). Kyoto Encyclopedia of Genes and Genomes (KEGG) and Gene Ontology (GO) enrichment analysis of differential expression genes (DEGs) were performed in Database for Annotation, Visualization, and Integrated Discovery (DAVID) bioinformatics software. Statistical and hypothesis testing was performed using GraphPad Prism 9. Graphical abstract and supplementary figure was created using BioRender.com.

For manuscripts utilizing custom algorithms or software that are central to the research but not yet described in published literature, software must be made available to editors and reviewers. We strongly encourage code deposition in a community repository (e.g. GitHub). See the Nature Portfolio guidelines for submitting code & software for further information.

Data

Policy information about availability of data

All manuscripts must include a data availability statement. This statement should provide the following information, where applicable:

- Accession codes, unique identifiers, or web links for publicly available datasets
- A description of any restrictions on data availability
- For clinical datasets or third party data, please ensure that the statement adheres to our policy

The authors declare that the data support the findings of this study are available within this Article, the Supplementary information or from the authors upon reasonable requests. Source data are provided with this paper. Sequencing data used in this study are available within the NCBI GEO database under the accession code GSE210373.

Human research participants

Policy information about studies involving human research participants and Sex and Gender in Research.

Reporting on sex and gender	Human OS resection tumor sections and primary tumors were used under IRB approval at JHU with a written informed consent for tissue banking. No sex and gender-based analysis were performed in this study.
Population characteristics	The diagnosis of high-grade conventional OS was verified by two independent bone pathologists .
Recruitment	N/A
Ethics oversight	Johns Hopkins University

Note that full information on the approval of the study protocol must also be provided in the manuscript.

Field-specific reporting

Please select the one below that is the best fit for your research. If you are not sure, read the appropriate sections before making your selection.

☒ Life sciences ☐ Behavioural & social sciences ☐ Ecological, evolutionary & environmental sciences

For a reference copy of the document with all sections, see nature.com/documents/nr-reporting-summary-flat.pdf

Life sciences study design

All studies must disclose on these points even when the disclosure is negative.

Sample size	Sample sizes were indicated in the legend of each Figure and Supplementary Figure. Eight replicates per group was calculated to provide 80% power to detect effect sizes of at least 1.5, assuming a two-sided 0.05 level of significance.
Data exclusions	Data were not excluded in the analysis.
Replication	All data types were independently reproduced with independent experiment numbers given in the Figure legends and Figures.
Randomization	All mice were randomly allocated into experimental groups. For cell based experiments in vitro seeded cell populations were randomly allocated to treatment groups prior to experimentation.
Blinding	For observer- based microscopy data assessment and collection, observers were blinded to sample identification Blinding was not relevant to the genomic data and automated image analysis because investigator's bias would not affect the data output For in vivo tumor size assessment, where analysis relied on observer based measurements, data collection was undertaken in a double blinded manner.

Reporting for specific materials, systems and methods

We require information from authors about some types of materials, experimental systems and methods used in many studies. Here, indicate whether each material, system or method listed is relevant to your study. If you are not sure if a list item applies to your research, read the appropriate section before selecting a response.

Materials & experimental systems

n/a	Involved in the study	
☐	☒	Antibodies
☐	☒	Eukaryotic cell lines
☒	☐	Palaeontology and archaeology
☐	☒	Animals and other organisms
☒	☐	Clinical data
☒	☐	Dual use research of concern

Methods

n/a	Involved in the study	
☒	☐	ChIP-seq
☒	☐	Flow cytometry
☒	☐	MRI-based neuroimaging

Antibodies

Antibodies used

Rabbit anti-CNTNAP4 Biorbyt orb544737
Mouse anti-Human Nuclei Millipore-Sigma MAB1281
Rabbit anti-CD31 Abcam ab28364
Rabbit anti-Ki67 Abcam ab15580
Goat anti-Mouse AF488 Abcam ab150117
Goat anti-Rabbit AF488 Abcam ab150077
Goat anti-Rabbit DyLight 594 Vector Laboratories DI-1594
Goat anti-Rabbit, HRP Invitrogen 32460
Rabbit anti-p44/42 MAPK (Erk1/2) Cell Signaling Technology 9102
Rabbit anti-Phospho-p44/42 MAPK (Erk1/2) Cell Signaling Technology 9101
Rabbit anti-FGF Cell Signaling Technology 61997
Rabbit anti-JNK Cell Signaling Technology 9252
Mouse anti- Phospho-JNK Cell Signaling Technology 9255
Rabbit anti-GAPDH Cell Signaling Technology 5174
Anti-mouse IgG, HRP-linked Antibody Cell Signaling Technology 7076
Anti-biotin, HRP-linked Antibody Cell Signaling Technology 7075
Anti-rabbit IgG, HRP-linked Antibody Cell Signaling Technology 7074

Validation

All primary antibodies used in this study were validated by the manufacture. Validation data / citations can be found on the manufacture website by searching the antibody catalog number provided in supplementary table S2 of our manuscript.

Eukaryotic cell lines

Policy information about cell lines and Sex and Gender in Research

Cell line source(s)

Human OS cell lines were procured from American Type Culture Collection (Manassas, VA), including 143B (ATCC®-CRL-8303™), Saos-2 (ATCC® HTB-85™), HOS (ATCC CRL-1543), KHOS/NP (ATCC CRL-1544), KHOS-312H (ATCC CRL-1546), and G-292 (ATCC CRL-1423).

Authentication

All the cell lines were purchased from ATCC and authenticated by ATCC

Mycoplasma contamination

Cell lines were not tested for mycoplasma contamination

Commonly misidentified lines
(See ICLAC register)

N/A

Animals and other research organisms

Policy information about studies involving animals; ARRIVE guidelines recommended for reporting animal research, and Sex and Gender in Research

Laboratory animals

All animal experiments were conducted according to approved protocols (MO21M112) of the Animal Care and Use Committee (ACUC) at Johns Hopkins University (JHU). NOD Scid mice (Stock No: 001303) were procured from The Jackson Laboratory (Bar Harbor, ME, USA).

Wild animals

N/A

Reporting on sex

For all 143B OS cell implantation, 8–10-week-old, male and female NOD Scid mice were used.

Field-collected samples

N/A

Ethics oversight

All animal studies were performed with institutional ACUC approval within Johns Hopkins University

Note that full information on the approval of the study protocol must also be provided in the manuscript.
